# DNA methylation marks associated with body composition in children from India and the Gambia: findings from the EMPHASIS study

**DOI:** 10.1186/s13148-026-02172-3

**Published:** 2026-06-19

**Authors:** Prachand Issarapu, Manisha Arumalla, Elie Antoun, Chiara di Gravio, Kate A. Ward, Caroline H. D. Fall, Andrew M. Prentice, Giriraj Ratan Chandak, Matt J. Silver, the EMPHASIS Study Group, Prachand Issarapu, Manisha Arumalla, Smeeta Shrestha, Sara Sajjadi, Sirazul Ameen Sahariah, Ramesh D. Potdar, Harsha Chopra, Harshad Sane, Meera Gandhi, Landing M. A. Jarjou, Ann Prentice, Sarah H. Kehoe, Stephen Owens, Andrew M. Prentice, Matt J. Silver, Giriraj R. Chandak, Caroline H. D. Fall

**Affiliations:** 1 Genomic Research on Complex Diseases (GRC Group), CSIR-Centre for Cellular and Molecular Biology, Hyderabad, India; 2 MRC Unit The Gambia at the London School of Hygiene and Tropical Medicine, Fajara, The Gambia; 3 London School of Hygiene and Tropical Medicine, London, UK; 4 Lightening Lives LLP, Hyderabad, India; 5 School of Medicine, University of Southampton, Southampton, UK; 6 MRC Lifecourse Epidemiology Centre, Human Development and Health, University of Southampton, Southampton, UK

**Keywords:** Epigenetics, DNA methylation, Body composition, EMPHASIS study, Epigenome wide association study (EWAS)

## Abstract

**Background:**

Differences in body composition during childhood can influence long-term health, with notable links to cardiometabolic disorders in later life. While genetic associations with body composition traits are well-studied, less is known about the role of epigenetic mechanisms, particularly in low- and middle-income countries where the burden of cardiometabolic disease is high. We investigated links between DNA methylation and three compartments of body composition: fat mass, lean mass, and bone measures using data from children enrolled in the *Epigenetic Mechanisms linking Pre-conceptional nutrition and Health Assessed in India and Sub-Saharan Africa* (EMPHASIS) study.

**Results:**

We conducted an epigenome-wide association study of 11 body composition traits assessed through dual-energy X-ray absorptiometry in children from India (mean [range] age = 5.8 [5–7] years; n = 686) and The Gambia (age = 9.0 [7–9] years; n = 284), with blood DNA methylation measured at ~ 800,000 CpGs sites on the Illumina EPIC array. Cohort-specific analysis identified 8 unique differentially methylated CpGs associated with traits across all three body composition compartments (*p* < 3.6 × 10^–8^), with none overlapping both cohorts. Cross-cohort meta-analysis revealed four CpGs associated with lean mass and bone area mapping to *SOCS3* and *ZBTB16*. Region-level analyses identified 29 differentially methylated regions (DMRs) in India and 18 in The Gambia. 29 DMRs were identified in the meta-analysis, 25 of which were not detected in either cohort individually. Many DMRs were associated with more than one body composition trait.

**Conclusion:**

We report novel DNA methylation signatures associated with body composition traits in children from two low- and middle-income countries. Identified loci map to genes linked to inflammatory signalling, energy metabolism and cellular stress response pathways, highlighting a potential role for epigenetic mechanisms in shaping early-life body composition.

**Supplementary Information:**

The online version contains supplementary material available at https://doi.org/10.1186/s13148-026-02172-3.

## Background

Body composition in childhood plays an important role in shaping long-term health outcomes [1, 2]. The three-compartment model, which evaluates fat mass (FM), fat-free mass or lean mass (LM), and bone mineral content (BMC), is used to estimate the body’s total functional capacity and overall health status [3]. Deviations in these measures can significantly increase the risk of various health conditions both in childhood and later in life [1, 4]. For example, excessive fat mass and insufficient lean mass during childhood are strongly linked to developing type 2 diabetes (T2D), hypertension and cardiovascular disease in adulthood [5–8], and obesity in childhood heightens the risk of several cancers, orthopaedic complications and cardiometabolic disorders [7, 9].

The prevalence of body composition-related disorders has been rising worldwide [10, 11], with large differences between high-income countries (HICs) and low- and middle-income countries (LMICs) [10]. Furthermore, LMICs such as India and The Gambia face a dual burden of malnutrition, with undernutrition coexisting with rising incidences of overweight and obesity. In the United States, 19.3% of children and adolescents aged 2–19 years were classified as obese in 2017–2018 [12]. In contrast, the prevalence of childhood obesity in India is estimated to be between 10 and 30%, varying by region [13], while in The Gambia, urban children show increasing trends of overweight and obesity [14].

Genome-wide association studies (GWAS) have helped to elucidate molecular mechanisms underlying body composition traits [15, 16], with genetic variants identified for key traits such as body mass index (BMI) [17], adiposity [18], and lean mass [19]. In addition to genetic influences, environmental factors including lifestyle, physical activity, and dietary practices play a significant role in shaping body composition [20], and there is growing interest in the potential for epigenetic mechanisms to mediate genetic and environmental effects on body composition and related traits [21–23]. For example, epigenome-wide association studies (EWAS) have identified associations between blood DNA methylation (DNAm) and adiposity [24], BMI [25], bone growth [26], obesity [27] and diabetes [28]. However, there is limited data from LMICs, where environmental and nutritional factors may have a greater impact on body composition [29].

The *Epigenetic Mechanisms linking Periconceptional Nutrition and Health Assessed in India and sub-Saharan Africa* (EMPHASIS) study [30] combines deep phenotyping with genetic and DNAm profiling in children from two LMICs, India and The Gambia. In our previous analyses, we reported EWAS findings linking DNAm with periconceptional maternal nutrition [31], cardiometabolic risk markers [32], and childhood height [29]. In this study, we aimed to identify genome-wide DNAm signatures associated with childhood body composition measures assessing lean, fat, and bone compartments. We additionally investigated whether these DNA methylation-phenotype associations were influenced by underlying genetic variation or nutritional interventions.

## Results

Baseline characteristics of the Indian (n = 686) and Gambian (n = 284) children are presented in Table 1. Gambian children were older and had higher BMI and absolute measures of lean mass, fat mass and bone area compared to Indian children. They also had greater bone mineral density and adiposity indices, whereas android fat was lower. Maternal age and BMI at recruitment were also higher in the Gambian cohort.

**Table 1:** Baseline characteristics of EMPHASIS cohorts analysed in this study

Child parameters	Indian (n = 686)		Gambian (n = 284)		*P*-value
Sex (F)	309 (44.3%)		129 (45.4%)		8.2 × 10^–1^
Age (yrs)	5.75	(5.63,5.98)	9.01	(8.63,9.22)	2.2 × 10^–16^
Height (cm)	109.4	(106.4,113.0)	130.4	(125.7,133.6)	2.2 × 10^–16^
BMI (kg/m^2^) *	13.3	(12.6,13.8)	14.2	(13.5,15.1)	2.2 × 10^–16^
Lean mass (kg)	12.7	(11.6,13.7)	18.3	(16.6,19.9)	2.2 × 10^–16^
Fat mass (kg)	2.24	(1.77,2.92)	4.69	(4.02,5.51)	2.2 × 10^–16^
Android fat (kg)	0.15	(0.12,0.20)	0.12	(0.10,0.17)	1.4 × 10^–9^
Gynoid fat (kg)	0.60	(0.49,0.74)	0.66	(0.48,0.83)	7.0 × 10^–3^
Lean mass index (kg/m^2^) *	10.6	(10.0,11.1)	10.9	(10.2,11.6)	2.1 × 10^–7^
Fat mass index (kg/m^2^) *	1.88	(1.48,2.45)	2.77	(2.41,3.25)	2.2 × 10^–16^
Body fat percentage (%) *	14.4	(11.6,17.9)	20.0	(17.4,22.6)	2.2 × 10^–16^
Bone area (cm^2^)	528	(469,583)	1107	(1025,1166)	2.2 × 10^–16^
Bone mineral content (g)	304	(262,349)	665	(586.30,744.60)	2.2 × 10^–16^
Bone mineral density (g/cm^2^) *	0.58	(0.55,0.60)	0.60	(0.56, 0.64)	4.0 × 10^–12^
N stunted (%)	105	(15.3%)	22	(7.7%)	1.9 × 10^–3^
N wasted (%)	255	(37.2%)	65	(22.5%)	2.19 × 10^–5^
*Maternal parameters*					
Age at recruitment (yrs)	25.0	(22.0,27.0)	30.0	(25.9,34.6)	2.2 × 10^–16^
BMI (kg/m^2^)	19.6	(17.7,22.5)	20.8	(19.3,23.08)	7.8 × 10^–10^
N Maternal intervention (%)	321	(46.8%)	135	(47.5%)	8.8 × 10^–1^

Median values are presented with interquartile ranges (IQR) in parentheses. n = number of participants included in the analysis; F = female; *P* values are from Mann–Whitney U tests for continuous outcomes and Chi-squared tests for categorical outcomes; * = derived measures.

**Fig. 1:**
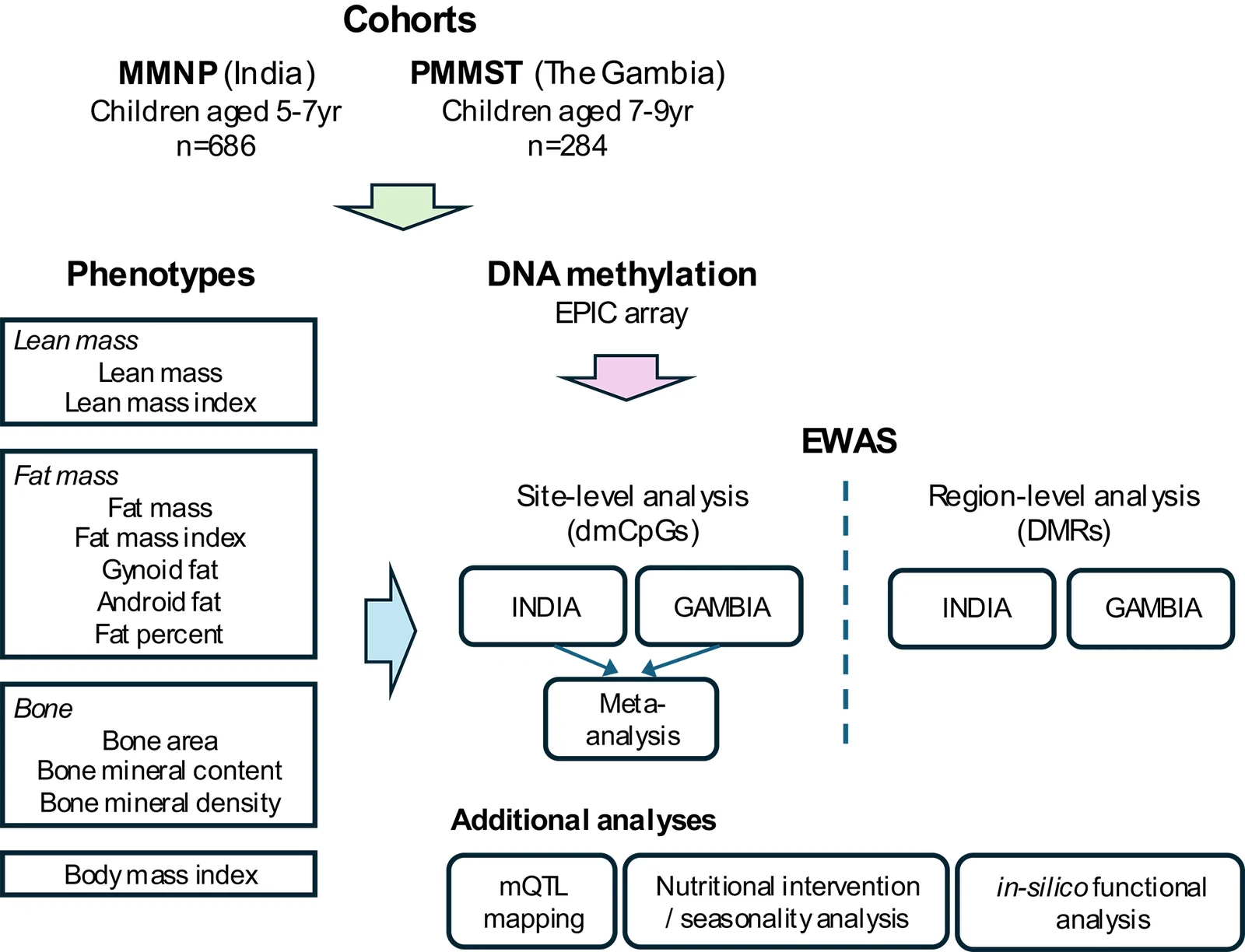
Schematic representation of study design. Body composition and body mass index phenotypes, together with genome-wide DNA methylation values were obtained for children from Indian and Gambian cohorts in the EMPHASIS study. Site-level and region-level associations were identified through epigenome-wide analysis. Additional analyses investigated genetic influences and in-silico functional effects. EMPHASIS: Epigenetic Mechanisms linking Periconceptional Nutrition and Health Assessed in India and sub-Saharan Africa; MMNP: Mumbai Maternal Nutrition Project; PMMST: Periconceptional Maternal Micronutrient Supplementation Trial; EWAS: Epigenome-wide association study; dmCpGs: differentially methylated CpGs; DMRs: differentially methylated regions; mQTLs: methylation quantitative trait loci; n = number of children

We conducted EWAS in each cohort to identify associations between peripheral blood DNAm measured on the Illumina EPIC array and 11 phenotypes covering the three compartments of body composition: lean mass (2 measures), fat mass (5) and bone measures (3) by dual-energy X-ray absorptiometry (DEXA), together with BMI (Fig. 1). EWAS were performed separately in the Indian and Gambian cohorts, followed by meta-analysis to identify shared associations. We then conducted regional analyses to identify differentially methylated regions (DMRs) within each cohort and across both cohorts combined. Further details of all analytical steps are provided in the Methods.

### Indian EWAS

**Fig. 2:**
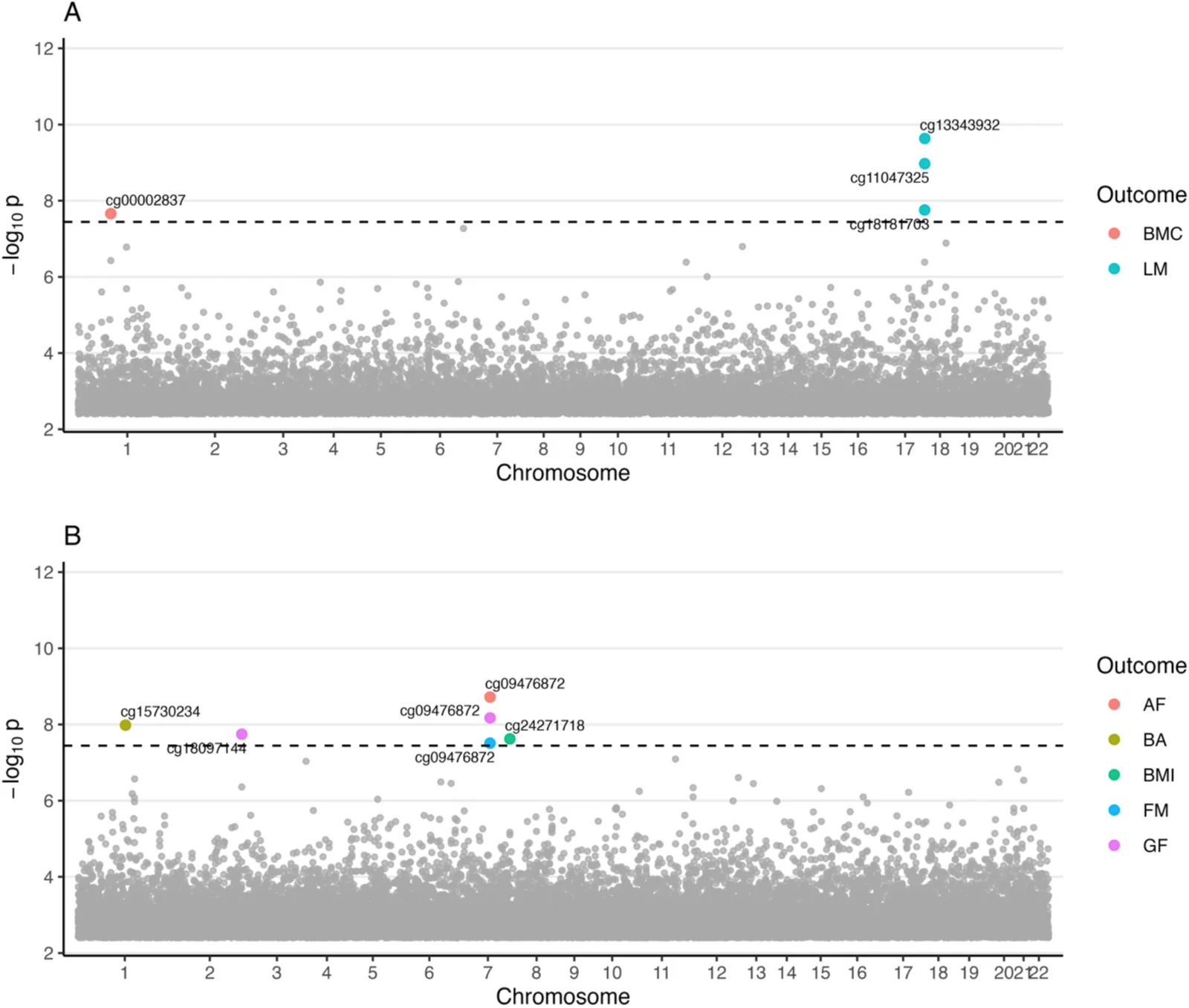
Identification of dmCpGs in Indian (**A**) and Gambian (**B**) cohorts. Significant CpGs are coloured according to outcome as indicated. The horizontal dashed lines represent the p-value threshold for significant methylome-wide associations accounting for multiple testing. AF = android fat; BA = bone area; BMI = body mass index; BMC = bone mineral content; FM = fat mass; GF = gynoid fat; LM = lean mass

Significant differentially methylated CpGs (dmCpGs) from the Indian cohort are presented in the top half of Table 2 and Fig. 2A.

**Table 2:** dmCpGs associated with body composition in Indian and Gambian children

Compartment	Outcome	CpG	Estimate*	95% CI	*P*-value	Chr	Pos	Gene name	Gene context
*Indian EWAS*									
Lean mass	LM	cg13343932	0.063	0.041, 0.084	2.1E-10	17	76,355,061	*SOCS3; LOC101928674*	CpG Island
		cg11047325	0.053	0.033, 0.072	9.9E-10	17	76,354,934	*SOCS3*	CpG Island
		cg18181703	0.077	0.047, 0.107	1.6E-08	17	76,354,621	*SOCS3*	N_Shore
Bone	BMC	cg00002837	−1.122	−2.053, −0.192	2.5E-08	1	44,513,358		5’UTR
*Gambian EWAS*									
Fat mass	FM	cg09476872	0.427	0.229, 0.625	3.1E-08	7	91,617,443	*AKAP9*	
	AF	cg09476872	0.019	0.007, 0.03	1.9E-09	7	91,617,443	*AKAP9*	
	GF	cg09476872	0.095	0.058, 0.132	6.7E-09	7	91,617,443	*AKAP9*	
		cg18097144	−0.02	−0.032, −0.007	1.8E-08	2	233,923,781	*INPP5D*	N_Shore; TSS1500
Bone	BA	cg15730234	6.816	4.074, 9.559	1.0E-08	1	101,759,418		
	BMI	cg24271718	0.483	0.257, 0.709	2.4E-08	7	151,402,023	*PRKAG2*	3’UTR

*Regression estimates represent a 1% change in DNA methylation associated with [estimate] units of change in the outcome. Significant associations with *p* < 3.6 × 10^–8^ accounting for multiple testing, are shown; 95% CI: 95% confidence interval, LM: lean mass (units: kg); FM: fat mass (kg), AF: android fat (kg), GF: gynoid fat (kg); BA: bone area (cm^2^); BMC: bone mineral content (g); BMI: body mass index (kg/m^2^). Genomic context annotations are based on ‘Gencode Basic V12’ and ‘UCSC CpG Island relationships’ from the Illumina EPIC manifest file. Pos = CpG location (hg19); Chr = Chromosome number; TSS1500 = 200–1500 bases upstream of transcription start site; TSS200 = 0–200 bases upstream of the transcription start site; UTR = untranslated region

#### Lean mass

Three dmCpGs were significantly associated with lean mass. These were located within the second exon of suppressor of cytokine signalling 3 (*SOCS3*) gene: a 1% change in DNA methylation was associated with a 0.06 kg in increase in lean mass at cg13343932 [95% confidence interval (CI): 0.041, 0.084; *p* = 2.1 × 10^–10^], and with 0.05 kg (95% CI: 0.033, 0.072, *p* = 9.9 × 10^–10^) and 0.077 kg (95% CI: 0.047, 0.11, *p* = 1.6 × 10^–8^) increase in lean mass for cg11047325 and cg18181703 respectively. No significant associations with lean mass index were observed.

#### Fat mass

There were no significant associations identified for the fat mass outcomes.

#### Bone measures

There was a single dmCpG, cg00002837, associated with a decrease of 1.12 g in bone mineral content per 1% increase in DNA methylation (95% CI: − 2.05, − 0.19; *p* = 2.5 × 10^–8^). No significant associations with bone area or bone mineral density were observed.

### Gambian EWAS

Significant dmCpGs from the Gambian cohort are presented in the bottom half of Table 2 and Fig. 2B.

#### Lean mass

No significant associations with lean mass or lean mass index were observed.

#### Fat mass

We found several significant associations with fat mass outcomes. A 1% increase in DNA methylation at cg09476872, mapping to the A-Kinase Anchoring Protein 9 (*AKAP9*) gene was associated with a 0.43 kg increase in fat mass (95% CI: 0.23, 0.63; *p* = 3.1 × 10^–8^). This CpG was also associated with a 0.02 kg increase in android fat (95% CI: 0.01, 0.03]; *p* = 1.9 × 10^–9^), and a 0.10 kg increase in gynoid fat (95% CI: 0.60, 0.13; *p* = 6.7 × 10^–9^). cg18097144, mapping to the Inositol Polyphosphate-5-Phosphatase D (*INPP5D*) gene, was also associated with gynoid fat, with a 1% increase in DNA methylation corresponding to a decrease of 0.02 kg (95% CI: − 0.03, − 0.01; p = 1.8 × 10^–8^). No associations were observed with Fat mass index and fat percent.

#### Bone measures

A single dmCpG, cg15730234 was associated with an increase of 6.81 cm^2^ (95% CI: 4.07, 9.56; *p* = 1.0 × 10^–8^) in bone area. No significant associations with bone mineral content or density were identified.

#### Body mass index

A 1% increase in DNA methylation at cg24271718 in the Protein Kinase AMP-Activated Non-Catalytic Subunit Gamma 2 (*PRKAG2*) gene was associated with an increase of 0.48 kg/m^2^ in BMI (95% CI: 0.26, 0.71; *p* = 2.4 × 10^–8^).

No dmCpGs overlapped both cohorts.

### Meta analysis

**Fig. 3:**
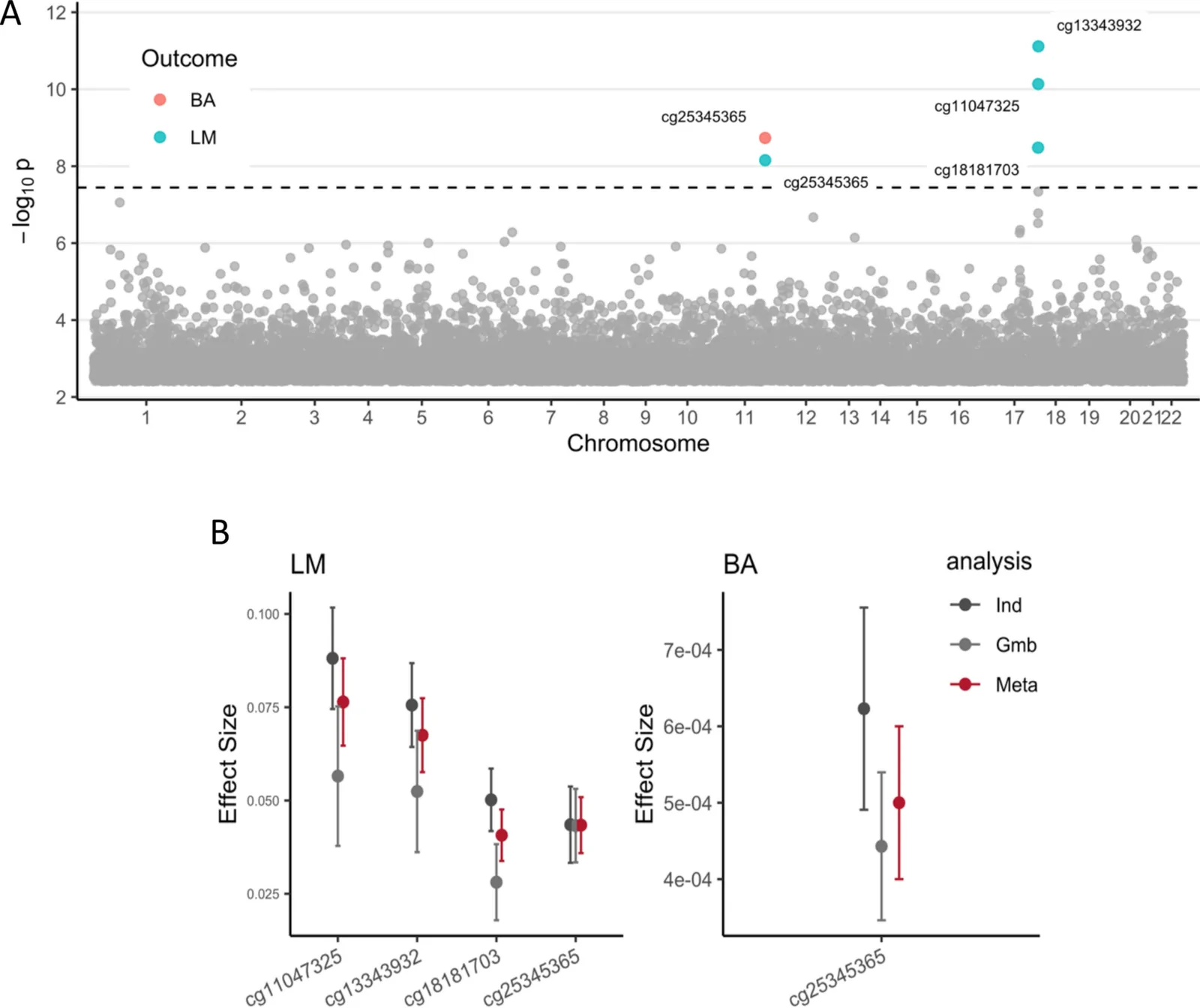
Meta-analysis of summary statistics from Indian and Gambian epigenome-wide association analysis. **A** Manhattan plot showing significant dmCpGs. These are coloured according to their corresponding outcomes. The horizontal dashed line represents the p-value threshold for significant methylome-wide associations accounting for multiple testing. **B** Forest plots showing effect sizes for significant CpGs in the individual cohort and meta-analysis. Error bars represent standard errors. BA: Bone area; LM: Lean mass; Ind: India; Gmb: Gambia; Meta: meta-analysis

Meta-analysis of summary statistics from individual cohort EWAS identified significant associations with lean mass and bone area adjusted for age and sex (Table 3, Fig. 3).

**Table 3:** Combined meta-analysis of cohort-level CpG associations

Outcome	Name	Estimate	StdErr	*P*-value	Direction	HetISq	HetPVal	Chr	Pos	Gene name	Genomic context
Lean mass	cg13343932	0.066	0.010	7.7E−12	++	19.9	0.264	17	76,355,061	*SOCS3; LOC101928674*	CpG Island
	cg11047325	0.055	0.009	7.3E−11	++	41	0.193	17	76,354,934	*SOCS3*	CpG Island
	cg18181703	0.079	0.014	3.3E−09	++	60.4	0.112	17	76,354,621	*SOCS3*	N_Shore
	cg25345365	0.045	0.013	7.0E−09	++	0	0.987	11	114,050,114	*ZBTB16*	
Bone area	cg25345365	2.939	0.718	1.8E−09	++	0.2	0.317	11	114,050,114	*ZBTB16*	

Cohort-level EWAS summary statistics from Indian and Gambian cohort were meta-analysed using an inverse variance fixed-effects model. Significant associations with *p* < 3.6 × 10^–8^ accounting for multiple testing, are shown. Regression estimates represent a 1% change in DNA methylation associated with [estimate] units of change in the outcome. HetISq and HetPVal values estimate the heterogeneity in effect sizes between cohorts. Genomic context annotations are based on ‘Gencode Basic V12’ and ‘UCSC CpG Island relationships’ from the Illumina EPIC manifest file. Pos = CpG location (hg19), StdErr = standard error, Direction = direction of effect in each cohort

Four dmCpGs were significantly associated with lean mass, three of which, cg13343932 (β = 0.07, *p* = 7.7 × 10^–12^), cg11047325 (β = 0.06, *p* = 7.3 × 10^–11^), and cg18181703 (β = 0.08, *p* = 3.3 × 10^–9^), mapped to the first exon of *SOCS3* gene on chromosome 17 and showed consistent direction of effect across both cohorts. The fourth dmCpG, cg25345365 (β = 0.05, *p* = 7.0 × 10^–9^), located within the zinc finger and BTB domain containing 16 (*ZBTB16*) gene, showed similar direction of effect in both cohorts. The same CpG at *ZBTB16* was also significantly associated with bone area (β = 2.9, *p* = 1.8 × 10^–9^), again showing consistent effect direction. There was no evidence of heterogeneity at any significant CpGs from the meta-analysis (all Het-I^2^ *p* > 0.05).

### Sensitivity analyses

#### Maternal micronutrient supplementation and Gambian season of conception

Mothers in both participating cohorts received pre- and peri-conceptional micronutrient supplementation [30]. In a previous report in the EMPHASIS study, we noted an effect of intervention on offspring DNA methylation at several loci [31]. We found no effect of the interventions on body composition-associated dmCpGs in either cohort (Supplementary Table 1). We additionally tested for an effect of season of conception in the Gambian cohort [31] and found no significant effect (Supplementary Table 2). Direct adjustment for estimated cell counts and batch variables resulted in attenuation of some EWAS-significant associations; however, all the loci remained statistically significant.

### Differentially methylated regions

We explored region-level DNAm associations for all 11 body composition measures, separately in Indian and Gambian cohorts, and across both cohorts combined in meta-analysis.

In the Indian cohort, we identified 29 significant DMRs associated with multiple body composition traits (Supplementary Table 3). 16 of these were associated with bone outcomes while 10 were identified with lean mass outcomes. Several regions overlapped across phenotypes, notably at *SOCS3* (3 CpGs, max *p* = 3.3 × 10^–8^), *TRIM72/PYDC1* (11 CpGs, max *p* = 1.5 × 10^–9^), *ASB3/ERLEC1* (5–11 CpGs, max *p* = 1.2 × 10^–9^), and *CDKL1* (6 CpGs, max *p* = 4.9 × 10^–8^), which were shared across lean mass, bone area, and bone mineral content. Additional overlapping DMRs were observed at *LTA* (4–6 CpGs, max *p* = 3.0 × 10^–8^) with lean mass and bone measures, and *ZNF772* (4 CpGs) showing opposite direction of association for lean mass index (*p* = 1.1 × 10^–8^) and fat percent (*p* = 3.6 × 10^–10^). Apart from these shared DMRs, there were multiple trait-specific DMRs identified in this cohort. DMRs mapping to *SOCS3* gene were overlapped with dmCpGs identified in the single-site EWAS analysis.

In the Gambian cohort, we identified 18 DMRs associated with multiple body composition traits (Supplementary Table 4). 8 of these were associated with fat mass, 3 with lean mass and 3 with bone outcomes. A shared DMR spanning *BPNT1/RNU5F-1* on chromosome 1 (4–6 CpGs; max *p* = 1.9 × 10^–8^) was associated with fat mass, fat mass index, fat percent and BMI. Additional shared regions included DMRs at *MTHFD1/MIR548AZ* (4 CpGs, max *p* = 1.1 × 10^–8^) and *HIF1A/HIF1A-AS1* (8 CpGs, max *p* = 3.6 × 10^–8^) associated with android fat and BMI, and at *LOC728264/MIR143HG* (max *p* = 1.9 × 10^–9^) associated with bone area, bone mineral content and lean mass. Other trait-specific DMRs included *PLAGL1/HYMAI, FUT4, MICAL1, KDM2A, NEK11/ASTE1* and *TCEB1* associated with android fat, bone density, BMI, fat mass index, gynoid fat, and lean mass index respectively.

There were no overlapping DMRs between Indian and Gambian cohorts.

Combining EWAS summary statistics in a meta-analysis, we identified 29 DMRs across the body composition traits (Supplementary Table 5). Of these, four regions overlapped with cohort-level findings: one overlapped only with Indian DMRs, corresponding to region at *SOCS3/LOC101928674*, and three overlapped only with Gambian DMRs, mapping to *MTHFD1/MIR548AZ*, and *TCEB1*. The remaining 25 DMRs that were specific to the meta-analysis spanned multiple body composition traits. Multi-trait DMRs mapping to *BAT2/SNORA38/PRRC2A*, *C20orf199/SNORD12*, *EFCAB4B*, and *STOX2* were associated with bone area, bone mineral content, bone mineral density, and lean mass, while a region mapping to *SAMD5* was associated with total fat mass and gynoid fat. Additionally, there were 12 trait-specific DMRs identified through meta-analysis.

A summary of outcomes with overlapping DMRs is presented in Supplementary Table 6 and Supplementary Fig. 1.

### Influence of genetic variants on body composition-associated dmCpGs

We investigated the potential influence of genetic variation on dmCpGs by conducting a methylation quantitative trait locus (mQTL) analysis on SNPs ± 1 Mb from the dmCpGs. This analysis was performed after SNP pruning to identify SNPs tagging linkage disequilibrium blocks. We found no significant associations with dmCpGs in either cohort after adjustment for multiple testing.

### Functional annotations

We explored the potential functional relevance of the significant dmCpGs by cross-referencing them with previously reported associations in the EWAS Catalog [33]. Of the 9 dmCpGs identified in our analysis, 8 had at least one recorded association in the EWAS Catalog (Supplementary Table 7). The dmCpGs annotated to *SOCS3* showed the highest overlap, appearing in more than 50 entries spanning inflammatory, metabolic and autoimmune phenotypes.

Pathway enrichment analysis using missMethyl [34] revealed no significant pathways after multiple-testing correction, and we also found no interpretable enrichment for cell type-specific epigenetic signals using eForge [35]. GREAT [36] identified several nominal enrichments under the region-based (binomial) test; however, none were supported by the gene-based (hypergeometric) test, indicating no robust ontology enrichment.

## Discussion

We investigated links between DNA methylation and body composition traits in children from two LMIC cohorts in the EMPHASIS study. Our study is notable for its comprehensive analysis of genome-wide DNAm signatures associated with four key DEXA measures: fat mass, lean mass, bone density and bone mineral content. Given the diverse genetic and environmental contexts between the Indian and Gambian populations, we conducted both cohort-specific analyses and meta-analysis to explore shared associations across populations. Through these complementary approaches, we identified 9 unique dmCpGs, most of which were trait specific. We also identified multiple trait-specific and overlapping DMRs associated with these traits providing potential insights into molecular mechanisms underpinning body composition traits in these understudied populations.

Our results highlight genes involved in pathways linked to inflammation, energy metabolism and growth regulation. Several identified genes play a role in immune-metabolic signalling that influences muscle and bone development. For example, *SOCS3*, a key regulator of the JAK/STAT pathway, modulates cytokine-driven processes in skeletal and muscle tissue and can influence insulin/IGF-1 signalling and bone remodelling [37–40]. In our analysis, *SOCS3* methylation was associated with lean mass and bone area, which is consistent with our previous observation that *SOCS3* methylation was linked to height in the same cohorts, with evidence of a causal effect in European populations [29]. Given the strong correlation between linear growth and body composition measures [41], it is plausible that *SOCS3* acts on a common growth/inflammatory axis impacting bone and muscle compartments. Signals at *LTA* [42, 43]*, SLC25A44* [44]*, PRDM15* [42, 43] and *P4HB* [45, 46] are implicated in inflammatory signalling and mitochondrial energy metabolism further supporting a potential role for these pathways in shaping childhood body composition.

A second group of genes related to cellular energy metabolism and muscle-fat homeostasis, suggesting that epigenetic variation in metabolic pathways may contribute to observed associations with DEXA-derived traits. *ZBTB16*, identified in the meta-analysis, regulates adipocyte differentiation and energy expenditure through *PPAR* signalling and thermogenic pathways [47, 48]. *PRKAG2*, encoding the γ2 regulatory subunit of AMP-activated protein kinase (*AMPK*), plays a central role in cellular energy sensing and glucose uptake [49, 50]. Signals observed at *HIF1A* and *MTHFD1* point towards additional pathways related to hypoxia responses and one-carbon/folate metabolism [51–53]. *HIF1A* regulates cellular adaptation to metabolic stress [51], while *MTHFD1* participates in folate-dependent biosynthetic and methylation reactions [53]. Although we did not test specific environmental drivers, these population-specific findings are consistent with the idea that DNA methylation-phenotype associations may be modified by local dietary patterns, seasonality, or morbidity profiles in different settings.

Taken together, these findings suggest that childhood body composition may be influenced by epigenetic variation in pathways linking inflammation, energy metabolism, and responses to environmental factors. Further replication of these findings, together with longitudinal and mechanistic studies, are needed to confirm these relationships.

Independent analyses of the Indian and Gambian cohorts revealed distinct epigenetic associations with no overlapping dmCpGs or DMRs. In the Indian cohort, many associated genes mapped to inflammatory or growth-related pathways, whereas in the Gambian cohort several genes mapped to metabolic stress or hypoxia-related pathways. This underscores the importance of conducting EWAS in diverse ancestries, as population-specific epigenetic signatures may arise from different combinations of genetic background, nutrition, infection exposure, and other early-life environments.

Several CpGs and DMRs showed overlapping associations across multiple traits at both the single-site and regional levels. The consistent direction of effects across these overlapping traits may reflect observed phenotypic correlations between lean, fat, and bone measures, or pleiotropic regulation through common pathways involved in growth, inflammation, and energy metabolism. Interestingly, DMR meta-analysis identified a number of multi-trait regions not detected within individual cohorts, highlighting the potential for this type of analysis to identify shared signals that are not apparent at the CpG level.

Childhood body composition predicts future risk for obesity, diabetes, and cardiometabolic disorders [1, 5, 7], suggesting that DNA methylation signatures identified in this study may have implications for long-term health. While our cross-sectional study cannot provide evidence of a mechanistic link, our findings highlight the importance of understanding how epigenetic mechanisms shape early growth and metabolic health across diverse environments, notably in LMIC populations where the dual burden of undernutrition and rising obesity is of increasing concern [13, 54].

The cohort-specific DNA methylation patterns observed in this study likely reflect broader environmental and socioeconomic differences between the Indian and Gambian settings. Alongside genetic background, factors such as maternal education and nutrition, childhood undernutrition, dietary diversity, and infection burden may all influence variation in body composition and epigenetic regulation in these children. These exposures, acting across early and later childhood, could contribute to the distinct DNA methylation-body composition associations observed between the two populations. Incorporating environmental and socioeconomic data in future analyses will be important to clarify how these context-specific influences shape epigenetic variation and growth outcomes.

While the study provides important insights, several limitations should be acknowledged. Our analysis of blood samples for DNAm analysis means that we were unable to analyse DNA methylation patterns in other tissues, such as adipose or muscle, which may be more relevant to body composition traits. Multi-tissue studies are needed to assess the tissue specificity of these associations. Additionally, the Indian cohort analysis was better powered with a larger sample size which could in part explain inter-cohort differences, and the greater overlap between Indian cohort and meta-analysis results. As with all observational studies, these associations do not establish causality and may be influenced by unmeasured factors such as genetic variation or maternal and child characteristics not captured in the EWAS models. Our findings require replication in independent cohorts and further functional validation to clarify their biological relevance. Finally, our analysis was restricted to the small fraction of the methylome captured by EPIC arrays, potentially missing important signals outside the assayed regions.

## Conclusion

Our study provides insights into the epigenetic regulation of body composition in children from two LMIC populations. We identified multiple CpGs and DMRs associated with fat mass, lean mass, and bone outcomes, most of which were cohort specific. Future research integrating genetic, environmental, and longitudinal data will help elucidate the mechanisms linking early-life DNA methylation patterns to body composition and later health outcomes.

## Methods

### Study cohorts

The cohorts included in this study are part of the EMPHASIS study (ISRCTN14266771; registration date: 18/05/2017; https://www.isrctn.com/ISRCTN14266771), which is a follow-up of children born to mothers who participated in two independent randomized controlled trials of peri-conceptional maternal micronutrient supplementation in two undernourished communities. The Mumbai Maternal Nutritional Project (MMNP: ISRCTN62811278) [30] was conducted from 2006 to 2012 in the slums of Mumbai, India, and enrolled 6513 women, resulting in 1562 live births. The Peri-conceptional Maternal Micronutrients Supplementation Trial (PMMST: ISRCTN13687662) [30] was conducted from 2006 to 2008 in West Kiang, The Gambia, and enrolled 1156 women, resulting in 376 live births. In the present EMPHASIS follow-up, children were recontacted at 5–7 years of age in India and 7–9 years in The Gambia. The analysis included the first 709 per-protocol children from India and 296 retraced children from the Gambian cohort. Peripheral venous blood samples were collected into EDTA vacutainers, stored at − 80 °C, and transported on dry ice for genomic DNA extraction using the Qiagen DNA Blood Midi Kit prior to DNA methylation profiling. Children with complete DNA methylation and DEXA data were included in the final analyses (India: n = 686; the Gambia: n = 284). Full details on the trial design, inclusion criteria, and follow-up procedures are provided in the study protocol and previous publications [30, 31].

### Body composition measures and pre-processing of phenotype data

Body composition was assessed by dual-energy X-ray absorptiometry using GE Lunar Prodigy scanners in India and GE Lunar iDXA scanners in The Gambia (GE Healthcare, Madison, WI, USA) [30]. Both sites followed standard instrument calibration and quality assurance procedures, including daily phantom scans, routine system calibration, and manufacturer-recommended quality checks to ensure measurement stability across time. Children were scanned using standard paediatric DEXA protocols, including removal of metal objects, and standardized supine positioning [30, 55, 56]. Scans were acquired using the paediatric software mode available on both systems.

Android and gynoid fat regions were defined using instrument-derived automated regions of interest, following GE iDXA/Prodigy conventions [56]. Bone area, bone mineral content, and bone mineral density were calculated for the whole body excluding the head, in accordance with International Society for Clinical Densitometry (ISCD) paediatric guidelines [56]. A total of 11 body size and composition phenotypes were analysed. Direct DEXA-derived measures included lean mass (LM), fat mass (FM), android fat (AF), gynoid fat (GF), bone area (BA), and bone mineral density (BMD). Derived measures included BMI (WT/HT^2^), lean mass index (LM/HT^2^), fat mass index (FM/HT^2^), total fat percentage (FM/WT × 100), and bone mineral content (BMC = BA × BMD). Poor-quality, movement-affected, or incomplete scans were excluded using standard DEXA quality control criteria prior to analysis [56]. All phenotype outcome variables were assessed for normality and log transformed wherever necessary (FM, FMI, FP, AF and GF). Residuals were generated by regressing each outcome against age and sex and correlations among the trait residuals are shown in Supplementary Fig. 2.

### Pre-processing of DNA methylation data

Detailed information on the data generation and pre-processing has been previously reported [31]. Briefly, genome-wide DNA methylation profiling for both Indian and Gambian cohorts was performed at a single laboratory (CSIR-Centre for Cellular and Molecular Biology, Hyderabad, India) using the Illumina MethylationEPIC BeadChip (‘EPIC array’). Samples from the two cohorts were processed in separate laboratory runs but were randomised across arrays and processing batches to minimise technical bias. Pre-processing and quality control were conducted, including checks for detection p-values, sex mismatches, and sample call rates, using the R package *meffil* [57] (https://github.com/perishky/meffil). Probes on sex chromosomes, cross-reactive probes, and probes with common SNPs at the CpG or single-base extension site were excluded. Data were normalized using *meffil’s functional normalization* method, which incorporates probe-type and dye-bias correction based on control probes [57]. Data from the Indian and Gambian cohorts were processed separately. Final analysed datasets comprised 686 samples with 803,210 probes for the Indian cohort and 289 samples with 802,283 probes for the Gambian cohort.

### Pre-processing of genotype data

Imputed genotype data were available for both the EMPHASIS study cohorts. Detailed information on the genotyping, quality control, and imputation has been reported previously [31]. Briefly, samples were genotyped using the Illumina Global Screening Array (GSA, v1.0) and underwent standard QC procedures, including exclusion of samples with low call rate (< 95%), sex or genotype mismatches, heterozygosity outliers, and related individuals. Variant-level QC included removal of SNPs with call rate < 98%, Hardy–Weinberg equilibrium *p* < 1 × 10^–6^, or ambiguous strand alignment. Data were pre-phased using SHAPEIT (version: v2.r900) and imputed with IMPUTE2 (version 2.3.2; https://mathgen.stats.ox.ac.uk/impute/impute_v2.html) using the 1000 Genomes phase 3 reference panel. Imputation and QC were performed separately for the Indian and Gambian cohorts. SNPs with a minor allele frequency (MAF) < 10% and an IMPUTE info score < 0.9 were excluded to ensure high imputation quality and reliability.

### Epigenome-wide association studies (EWAS)

#### Site-level differential methylation analysis

Epigenome-wide association analyses were carried out for each outcome using robust linear regression models implemented in the *ewaff* R package (https://github.com/perishky/ewaff). *ewaff* is specifically designed for EWAS and enables efficient robust regression analyses to account for potential heteroskedasticity and outliers across large numbers of CpG sites, with adjustment for known/hidden confounders using surrogate variables. Logit-transformed DNA methylation beta values (M-values) were regressed against body composition outcome residuals, adjusting for child age, sex, and the first 10 surrogate variables (SVs) derived from the 200 k most variable DNA methylation probes. SVs were used to account for batch effects and variability in estimated blood cell composition while retaining the effect from the outcome variable of interest. Supplementary Table 8 confirms strong associations between SVs, estimated blood cell proportions and technical batch variables, assessed using Spearman’s rank correlation for continuous variables (cell-type proportions) and the Kruskal–Wallis test for categorical variables (batch, Sentrix_ID, and Sentrix_position). Bacon [58] (https://www.bioconductor.org/packages/release/bioc/html/bacon.html) was used to correct for inflation in p-values, with residual inflation assessed through the genomic inflation factor (lambda; Supplementary Tables 9 and 11) and visualized via quantile–quantile (QQ) plots (Supplementary Figs. 3 and 4). Significant dmCpGs were defined as those passing a methylome-wide threshold *p* < 3.6 × 10^–8^, applied independently to each outcome. This threshold is based on the effective number of independent CpGs tested, accounting for the correlation structure among CpGs, as recommended by Saffari et.al (2018) [59]. For significant outcome-CpG associations, the models were re-run using DNA methylation beta values (instead of M-values) to generate interpretable regression estimates where effect size is proportional to a unit change in the outcome. These are reported in the main text.

#### Site-level differential methylation meta-analysis

Cohort-level EWAS summary statistics (effect sizes and standard errors from models using M-values) were combined using METAL (version: 2011-03-25; https://genome.sph.umich.edu/wiki/METAL) [60]. A fixed-effects inverse-variance model was used, as recommended for meta-analyses with a small number of cohorts, to obtain a pooled estimate of shared effects while assessing cohort-specific variation through the I^2^ statistic [61]. Genomic control correction was applied to each cohort prior to meta-analysis, and CpGs missing in one cohort were excluded. Significant CpGs were re-estimated using β-values for interpretability and are reported in Table 3. The same methylome-wide threshold used for the cohort-wise EWAS (*p* < 3.6 × 10^–8^) was applied for the meta-analysis, and both cohorts had identical covariates in EWAS models.

#### Region-level differential methylation analysis

DMRs were identified using the R package *dmrff* (v1.0.0) (https://github.com/perishky/dmrff) [62]. Analyses were conducted separately for each cohort using the same covariate-adjusted EWAS models. DMRs were defined as regions with ≥ 2 CpGs within 500 bp (maxgap) showing consistent direction of effect, and statistical significance was determined using the *dmrff* default Bonferroni correction at the region level (adjusted *p* < 0.05). Meta-analysis of DMRs was performed using the *dmrff.meta()* function combining EWAS summary statistics from both cohorts using the same regional criteria and significance thresholds.

### Sensitivity analyses

We performed a sensitivity analysis on dmCpGs identified in single cohort and/or meta-analysis to check for effects of maternal supplementation carried out in the original Indian and Gambian studies and Gambian season of conception [31]. A Bonferroni corrected *p*-value threshold of 0.05 was used in each cohort.

### Influence of genetic variation on DNA methylation signatures (mQTL analysis)

cis-mQTL analysis was conducted using the Gene-Environment-Methylation (*GEM*) package (v1.10.0; https://bioconductor.org/packages/devel/bioc/html/GEM.html) from R Bioconductor to assess SNP—DNA methylation associations. An additive allelic dosage (0,1,2) regression model adjusted for child age, sex and the first 10 PCs derived from the 200K most variable DNA methylation probes was used following the GEM pipeline. The PCs were evaluated for correlation with estimated cell counts and technical variables (Supplementary Table 11). This analysis was carried out on unique dmCpGs identified in single cohort and/or meta-analyses and SNPs within ± 1 MB of the dmCpGs that were ‘pruned’ to account for linkage disequilibrium between the SNPs. The pruning was carried out in plink2 (https://www.cog-genomics.org/plink/2.0/) using –indep-pairwise with the following parameters: window size = 1000 kb; step size (variant count) = 50; and r^2 = 0.2. This resulted in 971 and 3324 cis-SNPs in the Indian and the Gambian cohorts respectively. Significant cis-mQTLs were determined based on association p-values that passed a Bonferroni-adjusted significance threshold of *p* = 0.05/(n_SNPs_tested*n_CpGs_tested); where ‘n_SNPs_tested’ represents the number of cis-SNPs and ‘n_CpGs_tested’ is the number of unique CpGs tested in the corresponding cohort.

### Functional annotation

To investigate the functional relevance of identified dmCpGs and DMRs, we performed in silico analyses using publicly available databases. First, significant dmCpGs were cross-referenced against the EWAS Catalog (https://www.ewascatalog.org/) [33] to identify previously reported associations with relevant phenotypes. Next, gene set enrichment was assessed using missMethyl (https://www.bioconductor.org/packages/release/bioc/html/missMethyl.html; goregion() and gometh()), which accounts for probe-number bias on the Illumina EPIC array [34]. Both GO (Biological Process, Molecular Function, Cellular Component) and KEGG pathway enrichments were evaluated using default parameters, with significant pathways defined as those with FDR < 0.05. We additionally assessed regulatory enrichment using eFORGE v2.0 (https://eforge.altiusinstitute.org/) [35], which tests for tissue- and cell type-specific DNase hypersensitivity and histone mark enrichment (marginal *p* < 0.05, strict *p* < 0.01; proximity: 1 kb window). Region-based functional annotation was performed using GREAT v4.0.4 (https://great.stanford.edu/great/public/html/) [36], which assigns genomic regions to regulatory domains under Basal plus extension (5 kb upstream, 1 kb downstream, 1 Mb distal extension) and tests for ontology enrichment. Enrichment was evaluated using both binomial (region-based) and hypergeometric (gene-based) tests, with terms considered robust only if supported by both statistics at FDR < 0.05.

## Supplementary Information


Supplementary Material 1
Supplementary Material 2


## Data Availability

The data required for the results interpretation presented in this manuscript are provided within the manuscript and as supplementary material. DNA Methylation and genotype data are available on request to the corresponding authors. All software used in this study is open source, and links to the relevant packages are provided in the manuscript.

## Abbreviations

AF: Android fat
BA: Bone area
BMC: Bone mineral content
BMD: Bone mineral density
BMI: Body mass index
DEXA: Dual-energy X-ray absorptiometry
dmCpG: Differentially methylated CpG
DMR: Differentially methylated region
EWAS: Epigenome-wide association study
FDR: False discovery rate
FM: Fat mass
FMI: Fat mass index
GF: Gynoid fat
GO: Gene Ontology
GWAS: Genome-wide association study
KEGG: Kyoto Encyclopaedia of Genes and Genomes
LD: Linkage disequilibrium
LM: Lean mass
LMI: Lean mass index
mQTL: Methylation quantitative trait loci
