# Supplementary material for: DNA methylation marks associated with body composition in children from India and the Gambia: findings from the EMPHASIS study
Source: Clin Epigenetics. 2026 Jun 19;18:158. doi: 10.1186/s13148-026-02172-3 (PMC13528051; doi:10.1186/s13148-026-02172-3)
Supplement: Supplementary file 1 [file 13148_2026_2172_MOESM1_ESM.docx]

**Supplementary Figure 1.** UpSet plots showing the number of overlapping DMRs across outcomes in the Indian (ind), Gambian (gmb), and meta-analyses. Vertical bars represent the number of DMRs shared across specific outcome combinations, while horizontal bars indicate the total number of overlapping DMRs per outcome. Filled circles denote outcomes included in each intersection. Overlapping regions were defined based on genomic coordinates (chr, start-end) with at least 1bp overlap between the regions.

A)

B)

**Supplementary Figure 2.** Spearman’s correlation coefficients (r) and corresponding p-values between phenotype residuals used in A) Indian and B) Gambian EWAS analysis. BMI = Body Mass Index; FM = Fat Mass; LM = Lean Mass; AF = Android Fat; GF = Gynoid Fat; FMI = Fat Mass Index; LMI = Lean Mass Index; Fat% = Fat Percentage; BA = Bone Area; BMC = Bone Mineral Content; BMD = Bone Mineral Density.

**Supplementary Figure 3:** Quantile-Quantile (Q-Q) plots of EWAS analyses with genome-wide significant associations in the Indian cohort. Lambda indicates the genomic inflation factor. Triangles indicate data points outside the plot area. LM = lean mass; BMC = bone mineral content.

**Supplementary Figure 4:** Quantile-Quantile (Q-Q) plots of EWAS analyses with genome-wide significant associations in the Gambian cohort. Lambda indicates the genomic inflation factor. Triangles indicate data points outside the plot area. FM = fat mass; AF = android fat; GF = gynoid fat; BMI = body mass index; BA = bone area.
